# Multi-dimensional factors associated with unprotected anal intercourse with regular partners among Chinese men who have sex with men in Hong Kong: a respondent-driven sampling survey

**DOI:** 10.1186/1471-2334-14-205

**Published:** 2014-04-16

**Authors:** Yong Cai, Joseph T F Lau

**Affiliations:** 1 School of Public Health affiliated with School of Medicine, Shanghai Jiaotong University, No.227, South Chongqing Road, Shanghai 200025, PR China; 2 Centre for Health Behaviors Research, School of Public Health and Primary Care, Faculty of Medicine, The Chinese University of Hong Kong, 5/F., School of Public Health, Prince of Wales Hospital, Shatin, NT, Hong Kong 030000, PR China

**Keywords:** Men who have sex with men, Respondent driven sampling, Regular partner, Unprotected anal intercourse

## Abstract

**Background:**

The HIV prevalence and incidence among men who have sex with men (MSM) are high. Unprotected anal intercourse (UAI) with male regular partners (RP) is an important but under-emphasized risk behavior. The current study aimed to describe the prevalence of UAI with regular partner and the associated multi-dimensional factors with UAI among MSM in Hong Kong, China.

**Methods:**

Respondent Driven Sampling method was used to recruit participants. A total of 285 participants were recruited, of whom 211 (75.1%) had had anal sex with RP in the last six months and their data were analyzed in this report. Weighed data were presented and logistic regression methods were fit.

**Results:**

Participants’ high risk behaviors in the last six months included high prevalence of having had UAI with RP (45.8%), having had non-regular male sex partners (NRP: 27.3%) and UAI with such partners (18.9%). Adjusted for socio-demographic variables, factors associated with UAI with RP included: 1) substances use prior to having anal sex (65.7% versus 43.8%; AOR =2.36; 95% CI =1.07-5.18), 2) worry that condom use symbolizes mistrust (67.9% versus 44.3%; AOR = 2.91; 95% CI =1.19-7.10), 3) a lower perceived degree of the RP’s acceptance of condom use (91.7% versus 38.3%; AOR = 22.70; 95% CI =6.20-83.10), and 4) a higher level of impulsivity (61.1% versus 35.0%; AOR =4.02; 95% CI = 1.62-9.97). Two of these four variables, substances use (ORm = 2.28, 95% CI = 1.01-5.16) and perceived lower level of RP’s acceptance of condom use (ORm = 17.22; 95% CI = 5.06-58.62) were selected by the forward stepwise logistic regression model.

**Conclusions:**

MSM with RP in Hong Kong is subjected to high risk of HIV transmission. Risk factors of UAI are multi-dimensional and interventions need to take into account factors of structural, interpersonal and individual levels.

## Background

The human immunodeficiency virus (HIV) epidemic among men who have sex with men (MSM) in China is a public health threat as the HIV prevalence in this population has been high and keeps increasing [1-3]. According to a national survey in 2011, the average HIV prevalence among MSM in China was 4.9% [4]. In Hong Kong, the HIV prevalence among MSM was 4.08% in 2011 [5]. According to social marketing principles, the target population should not be seen as a homogeneous group and segmentation is required to improve effectiveness of interventions [6]. In this context, previous research has shown that the prevalence of unprotected anal intercourse (UAI) with male regular sex partners (RP) is significantly higher than that with male non-regular sex partners (NRP) among MSM [7]. UAI with RP among MSM contributes to the majority of the new HIV infections in the United States and in Lebanon [8,9]. Among the 61.4% of the MSM in China having RP, 78.6% also have had sexual intercourse with other male sex partners [10,11]. The proportion of new HIV infections attributable to sexual intercourse with RP, NRP and commercial sex partners (CSP) among MSM was 40%, 37% and 23% respectively in 2010 in China [10]. The proportion of new infections attributable to RP among MSM was 34% in 2002, and with an increase of 6%, infections attributable to RP among MSM started to prevail in the past decade in China [10]. It seems that HIV prevention in China are mainly venue-based (gay-saunas and bars) and hence focuses mainly on promoting safer sex with NRP [10]. Furthermore, a cohort study targeting MSM has shown that UAI with RP but not UAI with NRP predicts HIV sero-conversion [12]. Hence, the risk of HIV transmission via male RP among MSM is important but may have been overlooked.

Furthermore, our literature search did not locate any study reporting HIV intervention specifically promoting condom use with RP among MSM. To design such programs effectively, we need to identify multi-dimensional risk factors associated with UAI with male RP among MSM. Previous studies have shown that intimacy between heterosexual sex partners is associated with inconsistent condom use [13,14]. It is also seen that worry about breaching the trust is one of the obstacles hindering condom use between heterosexual regular partners [15]. A few studies have tested similar hypotheses among MSM indicating that trust, emotional connection with the partner, and intimacy were significant predictors of UAI [16-18]. Cognitive factors are known to be significantly associated with UAI among MSM [19] and HIV/ sexual transmitted infection (STI) risk perception is one of such factors [18-20]. Very few, if any, studies investigated specific assessment of the risk level of one’s RP among MSM [21]. This is important as monogamy does not seem to be the norm among MSM [22,23]. Assessment of the perceived risk of contracting HIV via the RP is equally important, as that may cause severe damage to the relationship with the RP, sense of anger, guilt and regret [24]. Previous studies targeting MSM that did not focus specifically on UAI with RP have reported significant contextual factors [19,25], one of which was the use of alcohol and substances prior to having anal sex with men [26-28]. Relatively few studies studied the importance of this factor with respect to UAI with RP among MSM [27,28].

Personality of the MSM is another important but often under-investigated risk factor [29]. Impulsivity usually displays behaviors characterized by little or no forethought, reflection, or consideration of the consequences; it has found to be significantly associated with various types of risk factors such as alcohol abuse [30]. Impulsivity is also likely to be related to HIV risk--particularly in incarcerated substance-abusing youth [31]. A study revealed that the relationship between intensity of methamphetamine use and total unprotected sex was strongest among participants who had higher levels of impulsivity among HIV-positive MSM [32]. According to our knowledge, no study has investigated the association between impulsivity and UAI with RP among MSM.

Respondent driven sampling (RDS) is a relatively new research method designed for collecting data from hard-to-reach populations [33]. It has been used in studies targeting MSM in China [34] and in other countries [35,36]. It requests participants to refer a fixed number of eligible prospective participants (usually three to five) to join a survey, while similar waves are repeatedly conducted until the required sample size is achieved. Incentives are usually involved when referrals are being made. Theoretically, an equilibrium state would be attained after several waves of recruitment, and unbiased statistical estimates approaching those obtained from random probability sampling would be obtained [33,35]. The RDS design is hence an improvement over the venue-based sampling method commonly used in HIV research targeting MSM [36,37].

This study investigated prevalence of UAI specifically with RP among Chinese MSM in Hong Kong, China. Multi-dimensional factors were investigated at different levels, including i) the individual’s background factors (socio-demographic variables such as age, educational level, marital status, and sexual orientation, etc.); ii) contextual factors (use of alcohol and substances prior to having anal intercourse); iii) interpersonal factors (intimacy with the RP, worry that condom use symbolizes mistrust, perceived degree of the RP’s acceptance of condom use, perceived HIV/STI risk level of the RP; and iv) personality factor (impulsivity). It is hypothesized that the aforementioned factors would be associated independently with UAI with RP among MSM.

## Methods

### Study population and sampling

The fieldwork was conducted during March 2009 through October 2009. Inclusion criteria were: age of at least 18 years old, residents of Hong Kong, and self-reporting having had anal sex with at least one man in the last 6 months. Prospective participants were informed that the survey was anonymous and would take 20 to 30 minutes to complete and its purpose is to improve health of the MSM in Hong Kong. Using RDS procedures, seven initial eligible participants served as seeds. In the first wave, the seven seeds each recruited a maximum of three participants whom similarly recruited a second wave of three or less participants. Each new potential participant obtained three coupons containing information about the study and the eligibility criteria for referrals. The RDS method usually can achieve equilibrium after five to six waves of referrals [38,39]. In this study, a total of 285 eligible MSM were recruited by the seven seeds. The sample size was hence 294. Equilibrium was reached at Wave 5, regarding distributions on socio-demographics (age, educational level, marital status, and employment status), sexual orientation, and history of HIV voluntary counseling & testing (VCT). The average network size was 21. Each participant received a monetary compensation of HK$ 100 (about USD 12) for the time spent.

Of all these participants, 211 (75.1%) of the participants had had anal sex with the RP, 131 (46.6%) had had anal sex with NRP; seven (2.5%) had had sex with CSP in the last six months. Those with RP were asked all the questions presented in this report, while those without RP (n = 74) were excluded from the analysis as they were asked a different set of questions (e.g. those related to different types of venue used to recruit those NRP and their social network) instead of questions included in this report, data of which were not described in this report. The purpose of this paper was hence to look specifically at UAI with RP and involved a sample of 211 MSM who had had anal sex with RP in the last six months. For those with more than one RP in the last six months, they were asked to make reference to the RP who had had the most recent episode of anal sex when answering the questions.

### Ethical considerations

Anonymous interviews were administered. The survey presented no more than minimal risk or harm to the participants. Participants were briefed in detail about the study. Verbal informed consent was then obtained before commencement of the interview. Written informed consent was not obtained to maintain absolute anonymity. Interviewers were however requested to sign a form pledging that they had made clear explanations to the participants and answered all questions before the participants signed the informed consent. Such a consent procedure has been used in similar studies [40,41]. The ethics committee of Chinese University of Hong Kong approved the recruitment procedure and the protocol described in this study.

### Measures

#### Background characteristics

Information on background characteristics was collected, including socio-demographic variables (age, educational level, marital status, and employment status), sexual orientation, history of HIV voluntary counseling & testing (VCT). Participants were asked whether they had had anal sex with two other different types of male sex partners (NRP, and CSP) in the last six months and whether they had had UAI with the RP and the two other types of male sex partners.

#### Contextual variables

Contextual variables included the use of alcohol and substances prior to having anal intercourse with the RP in the last six months.

#### Interpersonal-level variables

Four interpersonal-level variables were included in this study. First, intimacy with RP was assessed by four items (‘*You are satisfy with the relationship with your RP’, ‘You have a close relationship with your RP’, ‘You are equal in the relationship with your RP’ and* ‘*You can depend on your RP to solve private problem*’), each with three response categories (1-no, 2-not sure, 3-yes). A summative composite scale was formed with a factor analysis identifying one single factor (% variance explained = 54.63%; Cronbach’s alpha coefficient = 0.631, mean = 10.9 ± 1.8, range = 4-12). Higher scores indicated a closer relationship with the regular partner(s). Second, we assessed the degree of worry that condom use symbolizes mistrust by using a single item (*“Do you worry that condom use during anal sex symbolizes mistrust between you and your RP”*); the response was recode into a binary measure (yes and no/not sure). Third, perceived degree of the RP’s acceptance of condom use was assessed by using three questions (‘Y*ou can persuade your RP to use condom if you want to’, ‘Your RP supports the use of condom’ and ‘you and your RP have reached a consensus over condom use’*). The response categories were ‘yes’, ‘not sure’ and ‘no’. A composite scale was constructed by counting the number of item response reflecting perceived RP’s acceptance on condom use. Fourth, perceived HIV/STI risk level of the RP was gauged by asking three questions (‘*Your RP has ever been infected with STI’, ‘Your RP has high risk behaviors for contracting HIV’; ‘You have much chance of contracting HIV via your RP’*); these questions had three response categories (‘yes’, ‘not sure’ and ‘no’). A composite indicator variable was created by counting the number of item response reflecting perceived HIV/STI risk level of the RP.

#### Personality variable

The Chinese version of the 19-item Impulsiveness Scale of the I7 questionnaire was used as the measure of impulsivity [42]. Impulsivity is regarded as acting without first considering the possible consequences. I7 Impulsiveness Scale contains 19 items (e.g. *‘Are you an impulsive person’, and ‘Do you usually think carefully before doing anything?’*). Respondents answered the items by giving dichotomous (‘yes’ and ‘no’) responses. This scale assesses the personality trait of impulsivity with good reliability and validity [42,43]. The sum of 19 item scores was converted into a total score (Cronbach’s alpha coefficient = 0.71; mean = 5.3 ± 2.1; range = 0-19). Higher scores mean higher impulsivity.

#### Creation of categorical variables for the composite scores

For the composite variables of intimacy with RP and impulsivity were recode into three groups according to the percentiles of the scores (low: <25th percentile, middle: 25–75 percentiles, high: >75th percentiles).

### Statistical analysis

RDS data were analyzed by the respondent-driven sampling analysis tool (RDSAT). Weighed estimates based on RDS II estimators [44], taking into account the network size and recruitment pattern, were derived. Univariate odds ratios (ORu) and their respective 95% confidence intervals (CI) were presented. Adjusted odds ratios (AOR) were estimated. In addition, multiple forward stepwise logistic regression models were fit, using all variables with p < 0.05 in the univariate analysis as candidates and adjusted for background variables. Statistical significance was defined by p value <0.05. Other statistical analyses were conducted by using SPSS 16.0 for windows.

## Results

### Profile of participants

Of the 211 participants, weighted estimates showed that 53.6% of them were of age 20 to 30 years old; 60.1% had attained college or university education; 97.7% were never married; 78.6% were full-time workers. Of them, 87.3% expressed that they were gay; only 33.5% of them had ever taken up VCT in the last six months (Table 1).

**Table 1:** Crude and adjusted estimates of characteristics of MSM with RP in Hong Kong (N = 211)

	**n (Crude %)**	Weighted %* (95% CI)
**Socio-demographics**		
*Age group (years)*		
< 20	25 (11.8)	14.1 (9.5-18.8)
20-30	121 (57.3)	53.6 (46.5-60.7)
31-40	53 (25.2)	25.9 (21.1-30.8)
> 40	12 (5.7)	6.4 (2.8-9.3)
*Highest education level*		
Junior high school or less	9 (4.3)	6.7 (3.8-9.6)
Senior high school	58 (27.5)	33.2 (30.1-36.3)
College or university	144 (68.2)	60.1 (52.7-67.5)
*Current marital status*		
Single	206 (97.6)	97.7 (95.6-99.8)
Married	5 (2.4)	2.3 (0.2-4.4)
*Full-time employment*		
No	54 (25.6)	21.4 (13.6-28.9)
Yes	157 (74.4)	78.6 (71.1-86.4)
*Self-reported sexual orientation*		
Gay/Homosexual	183 (86.7)	87.3 (80.2-92.4)
Bisexual	28 (13.3)	12.7 (7.6-19.8)
*Ever tested for HIV in the last six months*		
No	129 (61.1)	66.5 (59.2-72.0)
Yes	82 (38.9)	33.5 (28.0-40.8)
**Sexual risk behaviors in the last six months**		
Have had anal sex both with RP and NRP		
No	155 (73.5)	72.7 (21.1-36.4)
Yes	56 (26.5)	27.3 (63.6-78.9)
Have had anal sex both with RP and CSP		
No	207 (98.1)	97.6 (95.8-99.0)
Yes	4 (1.9)	2.4 (1.0-4.2)
Have UAI with RP		
No	111 (52.6)	54.2 (43.6-62.3)
Yes	100 (47.4)	45.8 (37.7-56.4)
Had had UAI with NRP		
No	44 (78.6)	81.2 (43.6-62.3)
Yes	12 (21.4)	18.9 (37.7-56.4)

*Respondent driven sampling data were adjusted according to the network size and recruitment.

Patterns; CI, confidence interval.

### Sexual risk behaviors in the last six months

In the last six months, 27.3% had had anal sex with NRP and 2.4% of the sample had had anal sex with CSP during the same six-month period. The prevalence of UAI with male RP and NRP in the last six months was 45.8% and 18.9% respectively (Table 1).

### Item responses of the contextual, inter-personal and personality variables

Respectively 14.7% and 10.4% had had used substances and alcohol prior to having anal sex with the RP. The majority (close to 80% or more) of the participants gave item responses reflecting intimate relationship with the RP (77.1 to 91.9%) and perceived RP’s acceptance over condom use (87.9 to 94.1%) and did not worry that condom use symbolizes mistrust (85.1%; see Table 2). The majority (79.9%) of the participants’ RP had not contracted STI, while only about 20% of the participants clearly indicated that they did not perceived their RP as not having high risk behaviors for contracting HIV (77.2%) and that they did not perceived high risk of contracting HIV via their RP (20.7%; see Table 2).

**Table 2:** Crude and adjusted estimates of the frequency distributions of the contextual variables, interpersonal variables and personality variable among MSM with RP (N = 211)

	**n (Crude %)**	Weighted %* (95% CI)
**Contextual variables**		
*Substances use prior to having anal sex with the RP*		
No	176 (83.4)	85.3 (81.5-89.2)
Yes	35 (16.6)	14.7 (10.8-18.5)
*Alcohol use prior to having anal sex with the RP*		
No	189 (89.6)	89.3 (84.2-93.9)
Yes	22 (10.4)	10.7 (6.1-15.8)
**Interpersonal variables**		
*Intimate relationship with RP*		
You are satisfy with the relationship with your RP regular partner		
No	23 (10.9)	11.5 (9.9-12.8)
Not sure	3 (1.4)	1.9 (1.1-2.6)
Yes	185 (87.7)	86.6 (84.4-88.9)
You have a close relationship with your RP		
No	31 (14.7)	15.1 (14.2-16.0)
Not sure	3 (1.4)	1.9 (1.2-2.7)
Yes	177 (83.9)	83.0 (80.4-85.6)
You are equal in the relationship with your RP		
No	11 (5.2)	6.8 (4.7-8.9)
Not sure	2 (0.9)	1.3 (0.7-1.9)
Yes	198 (93.8)	91.9 (88.5-95.2)
You can depend on your RP to solve private problem		
No	44 (20.9)	21.7 (9.2-19.0)
Not sure	2 (0.9)	1.2 (0.7-1.7)
Yes	165 (78.2)	77.1 (73.8-80.5)
*Level of intimacy with RP*		
Low level (<P_25_ or <10)	28 (13.3)	14.8 (9.2-19.0)
Middle level (P_25–75_ or 10–11)	45 (21.3)	21.9 (17.7-26.8)
High level (>P_75_ or >11)	138 (65.4)	63.3 (58.4-67.5)
*Worry that condom use symbolizes mistrust*		
Yes	28 (13.3)	14.9 (9.4-19.5)
No	183 (86.7)	85.1 (80.5-91.6)
*Perceived degree of the RP’s acceptance of condom use*		
You can persuade your RP to use condom if you want to		
No or Not sure	18 (8.5)	9.8 (7.2-11.6)
Yes	193 (91.5)	90.2 (88.4-92.8)
Your RP supports the use of condom		
No or Not sure	24 (11.4)	12.1 (9.7-14.5)
Yes	187 (88.6)	87.9 (85.5-90.3)
You and your RP have reached a consensus over condom use		
No or Not sure	10 (4.8)	5.9 (4.1-7.7)
Yes	201 (95.3)	94.1 (92.3-95.9)
*Number item responses reflecting PR’s acceptance of condom use*		
<3 (lower acceptance)	36 (17.1)	19.4 (13.7-22.8)
=3 (higher acceptance)	175 (82.9)	80.6 (77.2-86.3)
*Perceived HIV/STI risk level of the RP*		
Your RP has ever been infected with STI		
Yes or Not sure	45 (21.3)	20.1 (17.6-24.3)
No	166 (78.7)	79.9 (75.7-82.4)
Your RP has high risk behaviors for contracting HIV		
No	52 (24.6)	22.8 (19.7-25.9)
Yes or Not sure	159 (75.4)	77.2 (74.1-80.3)
*You have much chance of contracting HIV via your RP*		
No	46 (21.8)	20.7 (18.0-24.8)
Yes or Not sure	165 (78.2)	79.3 (75.2-82.0)
Number item responses reflecting HIV/STI risk level of the RP		
>0 (higher HIV/STI perceived risk level)	67 (31.8)	30.3 (26.1-35.7)
=0 (lower HIV/STI perceived risk level)	144 (68.2)	69.7 (64.3-73.9)
**Personality variable**		
*I7 Impulsiveness Scale*		
Low level (<P_25_ or <4)	40 (19.0)	20.3 (16.4-25.1)
Media level (P_25–75_ or 4–6)	117 (55.5)	56.7 (51.9-60.6)
High level (>P_75_ or >6)	54 (25.6)	23.0 (19.1-27.8)

*Respondent driven sampling data were adjusted according to the network size and recruitment.

Patterns; CI, confidence interval.

### Factors associated with UAI with RP in the last six months

None of the background variables presented in Table 3 was significantly associated with UAI with RP in the last six months. Adjusted for the socio-demographic factors listed in Table 1, variables that were significantly associated with the UAI with RP included (Table 4): 1) substances use prior to having anal sex with the RP (65.7% versus 43.8%; AOR = 2.36; 95% CI = 1.07-5.18), 2) worry that condom use symbolizes mistrust between the participant and the RP (67.9% versus 44.3%; AOR = 2.91; 95% CI = 1.19-7.10), 3) a lower perceived degree of the RP’s acceptance on condom use during anal sex (91.7% versus 38.3%; AOR = 22.70; 95% CI = 6.20-83.10), and 4) a higher level of impulsivity (61.1% versus 35.0%; AOR = 4.02; 95% CI = 1.62-9.97). Two of these four variables related to substances use (ORm = 2.28, 95% CI = 1.01-5.16) and perceived lower level of RP’s acceptance on condom use during anal sex (ORm = 17.22; 95% CI = 5.06-58.62) were selected by fitting a forward stepwise logistic regression model (see Table 4). A number of variables were found to be non-significant, including alcohol use prior to having anal sex with RP, intimacy with the RP and perceived HIV/STI risk level of the RP.

**Table 3:** Associations between socio-demographic factors and UAI with RP among MSM (n = 211)

	**UAI with RP in the last six months**	
**Socio-demographics**	**n (row%)**	**ORu (95% CI)**
*Age group (years)*		
< 20	9 (36.0)	1.00
20-30	57 (47.1)	1.58 (0.65-3.86)
31-40	29 (54.7)	2.15 (0.81-5.72)
> 40	5 (41.7)	1.27 (0.31-5.19)
*Highest education level*		
Junior high school or less	3 (33.3)	1
Senior high school	25 (43.1)	1.52 (0.35-6.66)
College or university	72 (50.0)	2.00 (0.48-8.31)
*Current marital status*		
Single	98 (47.6)	1
Married	2 (40.0)	0.74 (0.12-4.49)
*Full-time employment*		
No	25 (46.3)	1
Yes	75 (47.8)	1.06 (0.57-1.97)
*Self-report sexual orientation*		
Gay/Homosexual	85 (46.4)	1
Bisexual	15 (53.6)	1.33 (0.60-2.95)
*Ever tested for HIV*		
No	65 (50.4)	1
Yes	35 (42.7)	0.55 (0.05-6.17)

ORu: univariate odds ratio; 95% CI: 95% confidence interval.

**Table 4:** Contextual, interpersonal and personality factors associated with UAI with RP in the last six months among MSM (n = 211)

		**UAI with RP in the last six months**		
	**n (row%)**	**ORu (95% CI)**	**AOR (95% CI)**	**ORm (95% CI)**
**Contextual level**				
*Substances use prior to having anal sex*				
No	77 (43.8)	1.00	1.00	
Yes	23 (65.7)	2.46 (1.15-5.26)*	2.36 (1.07-5.18)*	2.28 (1.01-5.16)*
*Alcohol use prior to having anal sex*				
No	92 (48.7)	1	1	NS
Yes	8 (36.4)	0.60 (0.24-1.50)	0.51 (0.20-1.32)	
**Interpersonal level**				
*Level of intimacy with the RP*				
Lower level (<P_25_ or <10)	13 (46.4)	1	1	NS
Middle level (P_25–75_ or 10–11)	20 (44.4)	0.92 (0.36-2.38)	0.79 (0.30-2.12)	
Higher level (>P_75_ or >11)	67 (48.6)	1.09 (0.48-2.46)	0.89 (0.38-2.11)	
*Worry that condom use symbolizes mistrust*				
No	81 (44.3)	1	1	NS
Yes or not sure	19 (67.9)	2.66 (1.14-6.19)*	2.91 (1.19-7.10)*	
*Number item responses reflecting HIV/STI risk level of the RP*				
=0 (lower perceived risk level of the RP)	65 (45.1)	1	1	NS
>0 (higher perceived risk level of the RP)	35 (52.2)	1.33 (0.74-2.38)	1.40 (0.76-2.57)	
*Number item responses reflecting PR’s acceptance of condom use*				
=3 (higher perceived degree of the RP’s acceptance of condom use)	33 (38.3)	1	1	
<3 (lower perceived degree of the RP’s acceptance of condom use)	67 (91.7)	17.7 (5.23-60.09)**	22.70 (6.20-83.10)**	17.22 (5.06-58.62)**
**Personality level**				
*I7 Impulsiveness Scale*				
Lower level (<P_25_ or <4)	14 (35.0)	1	1	NS
Middle level (P_25–75_ or 4–6)	53 (45.3)	1.54 (0.73-3.24)	1.84 (0.85-4.01)	
Higher level (>P_75_ or >6)	33 (61.1)	2.92 (1.25-6.82)*	4.02 (1.62-9.97)**	

AOR: adjusted OR, odds ratios adjusting for all socio-demographic variables; ORm: odds ratio obtained from forward stepwise multivariate logistic regression using significant variables of the univariate analysis as input; NS: not statistically significant in the multivariate analysis; *p < 0.05, **p < 0.01.

## Discussion

The sampled MSM with RP were at high risk of HIV/STD transmission. A previous study showed that UAI with RP but not that with NRP predicted HIV seroconversion [12]. In this study, prevalence of UAI with RP was high (45.8%) and was comparable to that reported in a study conducted in Beijing in 2011 (49.7%) [45], while it was lower than that obtained from studies conducted in Argentina (58.0%) and Kazakhstan (69.0%) [35,46]. Interventions focusing on UAI with RP among MSM are hence greatly warranted. Attention should also be given to sex with NRP among those with RP, as about 30% of the participants had had sex with NRP in the last six months and UAI was only involved. To socio-ecological model reminds us that we need to look at factors associated with UAI with RP among MSM at inter-personal, individual and structural levels [47].

Interpersonal factors need to be considered. Unlike results obtained from studies targeting heterosexual couples [13,14], quality of relationship was not associated with UAI with RP in this study. The majority of the participants reported a good relationship with their RP. Other factors on relationship between the MSM and his RP (e.g. trust) were however, important. Many participants worried that condom use with the RP would be interpreted as mistrust. In this study and previous studies conducted in homosexuals and heterosexual populations [13,14,16-18], the concern over mistrust was significantly associated with condom use during sexual intercourse. The data suggested that mistrust was common as one third of the participants believed that their RP was at high risk of HIV infection in the future or would pass HIV infection to them. The beliefs may be a reflection of the high prevalence of participants having both RP and NRP. In the context of mistrust but a good relationship, discussion about condom use might be avoided by the couple to minimize tension. A vicious cycle of perceived high risk of HIV transmission, mistrust, avoidance, and then perceived high risk might be created, which needs to be broken with improvement in communication skills through interventions. Furthermore, the absence of legal marriage to the same sex might further hinder MSM to form their families and remain monogamous. Structural factors therefore also need to be considered to improve the situation. Couple-based HIV interventions are warranted and have shown to be effective [48-50]. Difficulties include worry about separation, sexual abuse and sorry that such information would be a shock to the partner or would harm the partner [51].

Contextual individual-level factors such as psychoactive substance use prior to anal intercourse should be considered. In this study, the prevalence was about 15%. Similar to the results of other studies [25,28], the variable was significantly associated with UAI. In China, there is no substance use prevention program targeting MSM. Such programs are different from those targeting the general population as sexual orientation and risks need to be considered. Prevention and risk reduction programs are warranted to fill up the service gap. In this study, alcohol use was not significantly associated with UAI among MSM. The result was similar to that of a recent conducted among Hong Kong MSM [7]. However, mixed findings of the associations between alcohol/drug use and UAI among MSM [7,46,51] have been reported.

Impulsivity is another important individual-level factor. Impulsivity is defined as acting without first considering the possible consequences is another important individual-level factor [32]. In this study and others, impulsivity refers to relatively stable personality and its measure was not specific to sex-related behaviors. It is potentially related to sexual adventurism, which is usually defined as sexual risk taking and sexual sensational seeking [52-54]. UAI is therefore related to sexual adventurism and sensational seeking [52,54]. Although previous studies have reported a significant association between higher impulsivity and unprotected sex among adult young women, high school students [51,55], drug users [56] and substance-abusing youth [31], this is the first study finding out that impulsivity was significantly associated with UAI during sex with RP among MSM. A study has shown greater intensity of methamphetamine use and higher levels of impulsivity predicted more unprotected sex among HIV-positive MSM, which suggests that targeting impulsivity in interventions may help reduce sexual risk behaviors [32]. Some interventions have been successful to instill stronger self-control on alcohol abuse and drug addiction [57,58] and such components may need to be added to HIV interventions targeting this population of MSM.

The study has several limitations. The sample size was modest; some non-significant associations may be subjected to inadequate power. We used community based RDS method to increase representativeness. However, we were still unable to control for non-response bias. Non-random recruitment of peers by RDS method could influence the estimates in unknown ways [33]. The development of statistical analysis methods for RDS data was not readily available and risk factors analysis in the study was unweighted because the RDSAT does not entertain multivariate analyses. Data were self-reported and may involve reporting bias due to social desirability, while the survey was anonymous. Furthermore, the study did not involve HIV antibody testing and it is a limitation that we did not ask about HIV sero-status of the participants and their male regular sex partners. One of the reasons was that such data might not be reliable due to strong social desirability and may hence strongly be subjected to reporting bias. The HIV prevalence was about 4% in the study population, HIV testing rate (last six months) was rather low (about 33.5%) and we do not have data about disclosure of HIV status to male regular sex partners. We do not know about the prevalence of sero-discordant couples among our participants but we contend that the number of such cases would be small. Some of the scales were constructed for this study and had not been validated as there are few studies specifically focused on UAI with RP among MSM.

## Conclusions

In sum, the participants were at high risk of HIV/STI transmission as they have relatively high prevalence of having NRP and low prevalence of condom use with both RP and NRP. Participants in general, perceived that their RP might be at high risk of HIV/STI transmission. Issues arising from the relationship between such MSM and their RP, including feeling of mistrust associated with condom use, need to be addressed. Unlike the case of heterosexual risk factors, intimacy between the couple was however, not a significant factor. The issue on impulsivity has seldom been discussed in HIV prevention targeting MSM. It was a significant factor and further studies on interventions to increase self-control are warranted. Overall, HIV interventions aiming at reduction of UAI with RP among MSM need to consider multi-dimensional and socio-ecological factors at structural, interpersonal and individual levels. Lastly, the study used RDS method for data collection and hence to reduce sampling bias.

## Abbreviations

RDS: Respondent driven sampling; MSM: Men who have sex with men; HIV: Human immunodeficiency virus; UAI: Unprotected anal intercourse; RP: Regular partners; NRP: Non-regular male sex partners; CSP: Commercial sex partners; STI: Sexual transmitted infection; VCT: Voluntary counseling & testing; ORu: Univariate odds ratios; CI: Confidence intervals; AOR: Adjusted odds ratios.

## Competing interests

The authors declare that they have no competing interests.

## Authors’ contributions

Both authors contributed the design of this research. YC drafted the manuscript and has been involved in the interpretation of the data. YC and JL performed statistical analyses; YC and JL played a major role in the field survey. YC and JL made a substantial contribution to the interpretation of the data and had been involved in revision of the manuscript through all stages. Both authors read and approved the final manuscript.

## Pre-publication history

The pre-publication history for this paper can be accessed here:

http://www.biomedcentral.com/1471-2334/14/205/prepub
